# Problem-solving therapy for depression and common mental disorders in Zimbabwe: piloting a task-shifting primary mental health care intervention in a population with a high prevalence of people living with HIV

**DOI:** 10.1186/1471-2458-11-828

**Published:** 2011-10-26

**Authors:** Dixon Chibanda, Petra Mesu, Lazarus Kajawu, Frances Cowan, Ricardo Araya, Melanie A Abas

**Affiliations:** 1 Department of Psychiatry, University of Zimbabwe, Harare, Zimbabwe; 2 Counselling Services Unit, Harare, Zimbabwe; 3 Centre for Sexual Health and HIV Research, University College London, London, UK; 4 Department of Community Medicine, University of Zimbabwe, Harare, Zimbabwe; 5 School of Social and Community Medicine, Bristol, UK; 6 King's College London, Institute of Psychiatry, London, UK

## Abstract

**Background:**

There is limited evidence that interventions for depression and other common mental disorders (CMD) can be integrated sustainably into primary health care in Africa. We aimed to pilot a low-cost multi-component 'Friendship Bench Intervention' for CMD, locally adapted from problem-solving therapy and delivered by trained and supervised female lay workers to learn if was feasible and possibly effective as well as how best to implement it on a larger scale.

**Method:**

We trained lay workers for 8 days in screening and monitoring CMD and in delivering the intervention. Ten lay workers screened consecutive adult attenders who either were referred or self-referred to the Friendship Bench between July and December 2007. Those scoring above the validated cut-point of the Shona Symptom Questionnaire (SSQ) for CMD were potentially eligible. Exclusions were suicide risk or very severe depression. All others were offered 6 sessions of problem-solving therapy (PST) enhanced with a component of activity scheduling. Weekly nurse-led group supervision and monthly supervision from a mental health specialist were provided. Data on SSQ scores at 6 weeks after entering the study were collected by an independent research nurse. Lay workers completed a brief evaluation on their experiences of delivering the intervention.

**Results:**

Of 395 potentially eligible, 33 (8%) were excluded due to high risk. Of the 362 left, 2% (7) declined and 10% (35) were lost to follow-up leaving an 88% response rate (n = 320). Over half (n = 166, 52%) had presented with an HIV-related problem. Mean SSQ score fell from 11.3 (sd 1.4) before treatment to 6.5 (sd 2.4) after 3-6 sessions. The drop in SSQ scores was proportional to the number of sessions attended. Nine of the ten lay workers rated themselves as very able to deliver the PST intervention.

**Conclusion:**

We have found preliminary evidence of a clinically meaningful improvement in CMD associated with locally adapted problem-solving therapy delivered by lay health workers through routine primary health care in an African setting. There is a need to test the effectiveness of this task-shifting mental health intervention in an appropriately powered randomised controlled trial.

**Trial registration:**

ISRCTN: ISRCTN25476759

## Background

Mental disorders cause considerable suffering, disability and social exclusion in Africa, and are poorly recognised and undertreated [1,2]. In Zimbabwe, common mental disorders, such as depression mixed with anxiety, are found in over 25% of those attending primary health care services or maternal services, and in up to 30% of females in the community [3-5]. In the Zimbabwean Shona language, thinking too much (*kufungisisa*), along with deep sadness (*kusuwisisa*), and painful heart *(moyo unorwadza) *are terms in common use for emotional distress being close to European and American categories of common forms of depression and anxiety [3,6]

There is increasing evidence, mainly from other world regions but also rapidly growing evidence from within low income countries, that improving mental health is a low cost approach to improve quality of life and reduce disability [7,8]. Very little of this evidence, however, is from Africa. In Chile, low intensity low-cost treatments for depression have been integrated into primary health care [9]. These include, for example, psycho education, problem-solving therapy and self-help approaches [10,11]. Problem-solving therapy has been shown to be effective for depression and common mental health problems [12,13]. Previous attempts to deliver care for common mental disorders through primary care clinics in Zimbabwe although promising in the short-term had shown little long-term success due to reliance on overstretched nursing staff and lack of supervision [14]. In 2005, a government operation in *Mbare*, a township in Harare, resulted in many people becoming homeless or losing their livelihoods [15] and was perceived by the *Mbare *community to lead to high rate of emotional distress. Local stakeholders identified the need for a community mental health intervention. This had to be at no extra cost to the primary health care clinic, to utilise space outside the overcrowded clinic rooms, and to use methods already tested locally. A pilot intervention based on a problem-solving approach was identified [16]. It was suggested this be delivered by lay health workers via a 'Friendship Bench' (*Chigaro Chekupanamazano*) placed in the clinic grounds, and that a system of supervision and stepped care be part of the package. A team comprising psychologists, a primary care nurse and a psychiatrist adapted existing training materials on problem solving therapy [16,17] in the light of experience working with lay workers and general nurses in primary care. Adaptations included at least one home visit by the lay workers early in the therapy given it is normal practice for lay workers to visit clients in their homes, and encouraging clients to schedule some positive activities that really mattered to them to make life more rewarding. The training and the intervention were pre-tested in 5 lay workers and 143 primary care clients and found to be acceptable to them and to the lay workers. The aim of this pilot was to gather preliminary data on the effectiveness of this intervention and to see if the intervention would be feasible, and if so to gather ideas about how best to implement it on a larger scale.

## Methods

### Setting

*Mbare *is a high density suburb or township in the south of Harare. It is characterized by ethnic diversity and high unemployment with most residents relying on informal trading. The literacy rate is estimated to be over 90%. There are three government run Primary Health Care (PHC) clinics, staffed almost exclusively by general nurses, for a population of approximately 200 000. The study took place in all three clinics.

**Figure 1:**
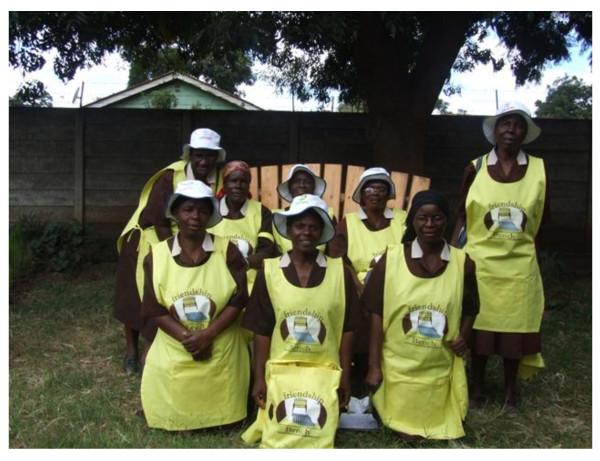
**Some of the lay health workers involved in the Friendship Bench project, sitting in front of one of the Benches**.

Twenty lay workers, locally termed health promoters, support the nurses at these three clinics. The lay workers are a respected group of primary health care providers, commonly referred to as *ambuya utano *(grandmother health provider) (Figure 1). In *Mbare*, all lay workers are female, literate, have at least primary school education, and have lived locally for at least 15 years. Their mean age is 58 years. Their main role is in community health outreach, which includes supporting people living with HIV/AIDS and Tuberculosis by providing individual and family support (practical, psychological and spiritual) and encouraging medication adherence. They also deliver community health education and promotion e.g. through encouraging immunisation and methods to control disease outbreaks. Lay workers report weekly to the environmental health officer and a nurse-manager. The lay workers cover geographical patches, which are sections of the community demarcated by the City of Harare according to street grids. Each geographical patch has approximately 3000 inhabitants. Ten lay workers were selected at random for this pilot: three from two of the clinics and four from the largest clinic.

### Participants

Inclusion criteria: aged 18 and over; residents of geographical patches in *Mbare*, Harare, covered by the ten selected lay workers; score > 7 on Shona Symptom Questionnaire screen for common mental disorders. Exclusion criteria: requiring acute medical attention such that they cannot participate; severe psychiatric symptoms and/or risk to self or others requiring specialist referral as assessed by primary care research nurse

Ethical approval was obtained from the Medical Research Council of Zimbabwe and written informed consent was sought from all participants. The study was registered as a non-controlled trial http://www.controlled-trials.com/ISRCTN25476759

### Recruitment

We aimed to recruit from the clinic staff, from the community, and from the lay workers themselves. The psychiatrist (DC) and psychologists (PM, KJ) presented to the clinic nursing staff and to all 20 lay workers the rationale for the project and referral methods to the friendship Bench. Notices written in the local vernacular language explaining the location and uses of the benches were placed at six different points within the entrance hall and waiting area of each clinic.

The lay workers introduced and publicised the Friendship Bench to the community through community stakeholders' meetings and during visits to people's homes, churches, schools and police stations. They introduced it as an adjunct to their normal daily community health outreach activity. They described the Friendship Bench approach as aimed at addressing common mental health issues such as *kufungisisa *(thinking too much) as a result of, among other things, HIV infection, AIDS, domestic violence, family sickness and poverty.

Clients were either referred or could self refer to the Friendship Bench, which was available Mon-Friday 9.00 am to 12.00 pm at each clinic. Those referred or who self-referred were directed by nursing or reception staff to sit on the Friendship Bench which in each clinic was a large wooden bench located under a tree within sight of the lay workers' office. One duty lay worker was responsible for the Bench each day on rotation and would approach the Bench after a potential client sat on it. The duty lay worker was responsible for collecting data on inclusion criteria including residential and basic demographic information and on psychological symptoms using the Shona Symptom Questionnaire (SSQ) [4]. She also gathered information on recent stressors using a brief life events screen based on one used previously in Harare [18]. Everyone was offered some education, advice and often sign-posted to support services. Those meeting inclusion criteria were referred to a research nurse for assessment of risk to self or to others (e.g. suicidal ideation, history of deliberate self harm, very severe symptoms). She referred those excluded on these grounds to the visiting psychiatrist (DC). She invited those meeting eligibility criteria to participate in the pilot and took written informed consent. She then referred them back to the lay worker who made arrangements for their first Friendship Bench session within 2-5 days with a lay worker that covered their geographical patch.

### Outcome measure

The main outcome measure was the Shona Symptom Questionnaire (SSQ). The SSQ is a 14-item screening tool for common mental disorders, integrating local idioms and internationally recognised items for emotional distress. It was developed and validated in Zimbabwe using exemplary cross-cultural methods [4]. It is self-administered and has a reliable internal consistency (r = 0.85) and satisfactory sensitivity and specificity, with a score of > = 8 being the cut-point. It is based on a yes/no response and asks about symptoms such as thinking too much, failing to concentrate, work lagging behind, insomnia, suicidal ideation, unhappiness and so on, over a 1 week period. All participants were approached six to eight weeks after their first treatment session to complete a self-administered SSQ which was collected by the research nurse in the absence of the attending lay worker.

### The Intervention

The intervention consisted of brief individual talking therapy based on problem-solving therapy delivered by a lay worker. Most sessions took place sitting on a bench termed "The Friendship Bench" (*Chigaro Chekupanamazano*). The Friendship Benches were made for the project by local craftsmen (see Figure 1). They are located within the grounds of each of the three participating clinics in a discrete area under the trees in the clinic gardens.

Table 1 shows the activities involved in the delivery of the Friendship Bench. The lay worker would initially explain to all participants how to self-administer the screening tool, the Shona Symptom Questionnaire. Problem-solving therapy (PST) included identification and exploration of problems, and identification and implementation of solutions, based on prior principles [19]. Our PST was a locally developed seven-step plan previously used in partnership with government, lay and traditional care providers [16]. Up to a maximum of 6 sessions on the Bench were offered with the second session taking place at the client's home and sometimes also one of the later sessions. Those most in financial need were referred to two local income-generating projects (peanut butter making; recycling). The problem solving therapy was enhanced with a component of activity scheduling in that clients were also encouraged to carry out activities that really mattered to them to make life more rewarding. Home visits included prayer. Prayer was already a well recognised part of the support provided by LW in their community health outreach role in *Mbare*, which has a 98% Christian population with more than 70 Christian faith groups. On average each prayer lasts 15-30 minutes and is delivered by one lay worker together with the family. The aim of the prayer is to comfort the sick and the family. The use of prayer in formal gatherings related to health is a common practice in Zimbabwe. The existing prayer format used prior to the introduction of the Friendship Bench was incorporated in the six sessions without any alterations.

**Table 1:** Features of Friendship Bench Intervention

Theoretical basis	Based on problem-solving therapy
**Delivering agent**	Lay health workers (Health promoters). Mean age 58 sd 8.3, all female, mean years of education 8 sd 3.3, previous training in home based care for people living with HIV & AIDS, in community follow-up of persons on TB treatment and in delivering community health education and promotion e.g. through encouraging immunisation and methods to control disease outbreaks

**Structure of intervention**	Six weekly sessions of 30-45 minutes delivered through the Friendship Bench over six weeks, including one or two home visits.

**Structure of sessions and areas covered**.	Session 1. **Problem identification**: A) Share Shona Symptom Questionnaire (SSQ) information with client, explain symptoms in relation to *kufungisisa*, B) Actively listen to clients story, identify problems raised, clearly define problem/s. **Problem exploration**: C) understand the story, help client prioritise problems, D) brainstorm practical/feasible solutions, outline the options available, encourage client to think over solutions. Session 2. E. Home visit before next session/prayer with family. Session 3. F) Summarise session 1, prioritise problems then identify solution that is feasible, provide information on referral where necessary. **Action plan: **G) Zero in on a specific solution, focus on what client wants to do, H) How, when, what assistance is needed? Referral if necessary e.g. to income generating program. I). Identify activities the person used to find rewarding and which matter to them and encourage these Session 4. J) Brief review of session 3, **Implementation: **K) How will it be done? Motivate; homework, Refer if appropriate to agency or support group. Session 5) Home visit. **Follow up: **L) What has been achieved? What were/are the obstacles if any? Go back to session 3 and 4. Session 6: M) Reinforce sessions 3, 4 and 5. What has been achieved, repeat SSQ score. N) No improvement refer to nurse counsellor

**Tools**	Multiple Symptoms Card seven step treatment plan, Referral manual

**Training**	Two-week training before onset of Friendship Bench. Ongoing training every two weeks for the first six months; thereafter monthly.

**Supervision**	Weekly one-hour group supervision by a general nurse with training in counselling. Group supervision from a clinical psychologist one hour every two weeks; group supervision by a psychiatrist every four weeks, 45 minutes.

### Training, selection and supervision of facilitators

All 20 lay workers were trained.

We provided an 8-day training run by two clinical psychologists (PM and LK), a general nurse trained in systemic counselling (ST) and a psychiatrist (DC). This covered didactic lectures on common mental disorders (CMD), including *kufungisisa *(thinking too much) but particularly focussed on skills to identify CMD using the Shona Symptoms Questionnaire [4], and to manage CMD using simple psycho-education and problem-solving therapy [16-19]. Lay workers then took part in two days of pre-testing including screening, identification, and referral processes within the clinic, and referral of 'red flags' (critical case-situations such as suicidal risk). We made use of practise with clients on the Friendship Bench and in clients' homes'. We developed a client referral manual, which included a list of NGO's, private and public institutions, and church organizations to be used by lay workers or patients.

Ten lay workers were selected at random for the pilot: three from two of the clinics and four from the largest clinic.

A daily peer-support group for lay workers was introduced. The peer group meetings were facilitated by one of the lay workers who would then present during weekly group supervision where all lay workers participated. A clinic staff nurse trained in counseling provided weekly group supervision at the largest clinic. The clinical psychologist and the psychiatrist provided further supervision every fortnightly and monthly, respectively.

We developed a brief 6-item questionnaire with a 4-point Likert scale for the lay workers to evaluate the PST intervention. For instance, we asked them to rate the ease with which they had learned the problem-solving therapy approach, the ease with which they delivered the intervention and the proportion of clients who appeared to benefit from the PST approach. We asked the lay workers to complete this once 6 weeks after the study has begun. We also carried out one focus group with 6 of the 10 workers and asked them to describe their experiences of delivering the intervention. Their responses were recorded in writing and analysed for content and themes by two of the authors.

### Data analysis

Descriptive statistics (means and standard deviations and proportions) were estimated for those who participated, who declines, who were lost to follow, and who were excluded due to psychiatric risk. We used t-tests and regression models to test changes in SSQ scores before and after completion of the treatment, adjusting for SSQ scores at baseline. Data were entered and analysed using EpiInfo 2002 and STATA 10.0 (Release 10, College Station, TX: Stata Corporation. 2003) after range checks and double entry of all questionnaires.

## Results

### Recruitment and attrition at follow-up

**Figure 2:**
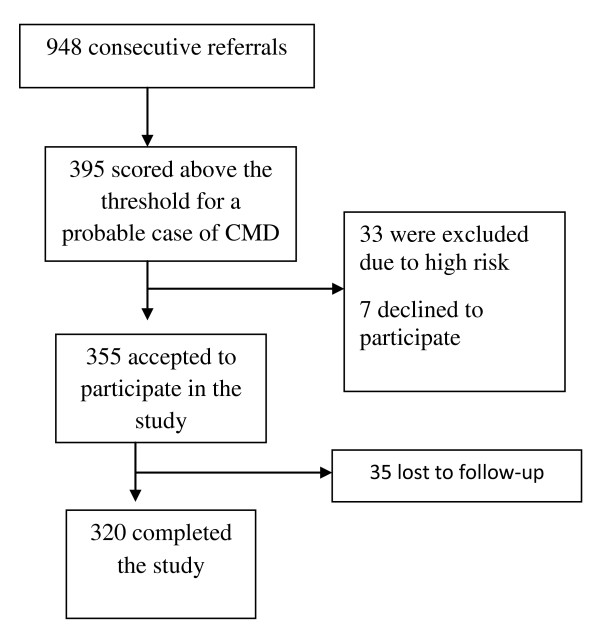
**Flow diagram of recruitment into the study**.

Between July and November 2007, 948 persons visited the Bench. Of these 948 persons who visited the Bench, 395 (42%) scored above the cut-point of the Shona Symptom Questionnaire (SSQ). Among these, 33 (8%) with a mean SSQ score of 11.8 (sd 1.2) were excluded from the pilot study due to being severely depressed and/or suicidal and were referred to the psychologist or psychiatrist (see Figure 2). Of the 362 invited to take part, 2% (7) declined and 10% (35) were lost to follow-up leaving an 88% response rate (320 participants). Of the 395, 188 (48%) presented with an HIV-related problem of whom 166 (88%) participated.

Table 2 shows the characteristics of the 395 who scored above the cut-point of the SSQ, according to whether or not they entered the study. Participants were more likely to be female. More of those who participated were female and married (70% female, 57% married) compared to those who declined (42% female, 43% married) or who were those lost to follow-up (40% female, 41% married). Those with less than eight years of education were more likely to be lost to follow-up than to participate. The primary reasons presented for visiting the Bench among those who participated were HIV-related, somatic complaints and domestic violence.

**Table 2:** Characteristics of individuals scoring above the cut off score (> = 8) at baseline on the Shona Symptom Questionnaire (n = 395)

	Declined N = 7	Participated N = 320	Loss to f/up N = 35	Excluded N = 33
**Mean Shona Symptom Questionnaire score **(sd)	10.8 (2.5)	11.3 (1.4)	10.2(1.4)	11.8 (1.2)

**Mean age **(sd)	26 (6.5)	27(7.0)	27(5.6)	29(6.0)

**Female **(%)	3(42%)	223 (70%)	40(40%)	23(69%)

**Referred by**				

Clinic staff	3(43%)	113(35%)	11(31%)	15(45%)

Lay Workers	1 (14%)	75(24%)	9(26%)	10(30%)

Friend/relative	1(14%)	41(13%)	4(11%)	3(9%)

Self referral	2(28%)	40(12%)	2(6%)	4(12%)

Other referrals	0 (0%)	51(16%)	9(26%)	1(3%)

**Education **≤ 7 years	1(14%)	38(12%)	8(23%)	5(15%)

**Marital Status**				

Married	3(43%)	182(57%)	14(41%)	16(48%)

Widowed	2(28%)	90(28%)	84(24%)	6(18%)

Divorced/single	2(28%)	48(15%)	9(25%)	11 (34%)

**Primary reason for visit**				

Somatic complaint	1(14%)	58(18%)	3(8%)	7(21%)

HIV-related	3(43%)	166(52%)	9(26%)	10(30%)

Domestic violence	1(14%)	54(17%)	11(33%)	7(21%)

Other	2(29%)	42(13%)	12(34%)	9(28%)

Most of those who participated were referred to the Friendship Bench by clinic staff (35%) and lay workers (24%). Other common forms of referral were: friend/relative (13%), self-referral (12%) or police (9%).

### Psychological symptoms scores before and after the six-week intervention period

All participants completed a minimum of 3 sessions over a six week period with 20%, 30%, 21% and 30% completing 3, 4, 5 or 6 sessions respectively.

The mean SSQ score for the 320 cases was 11.3 (sd 1.4) before treatment. After receiving between 3 to 6 sessions the mean score dropped by 4.8 points to 6.5 (sd 2.4) [t = 13.6 (p = 0.0087)]. For those completing 3 or more sessions, 66% recovered to below case level on the SSQ at 6-8 weeks

Table 3 shows the drop in SSQ scores according to the number of sessions attended, adjusting for baseline SSQ score. The more sessions attended the larger the drop in SSQ scores with a drop of more than 3 points observed among those who attended all six sessions.

**Table 3:** Drop in Shona Symptom Questionnaire score at 6 weeks after starting the intervention according to number of sessions attended

Number of sessions attended	N (%)	Regression Coefficients	95% Confidence Intervals	P value
**3**	63 (20%)	0.31755	-0.13 to .019	0.698

**4**	96 (30%)	-.780712	-1.46 to -0.11	0.024

**5**	66 (21%)	-1.758163	-2.49 to -1.03	0.000

**6**	95 (30%)	-3.258465	-3.93 to -2.59	0.000

**Total**	320			

Regression models adjusted by Shona Symptom Questionnaire score baseline scores

### Lay workers evaluation

Nine of the ten lay workers rated themselves as very able to deliver the PST intervention. All of them rated at least half of their clients as benefiting from PST with 7/10 rating 'more than half' of their clients benefiting from the intervention. Themes emerging from the focus group suggested that the lay workers viewed effective ingredients of the Friendship Bench to include:

a) Their position of trust in the community-clients viewed them as wise and confidential. The clients viewed them as 'persons who would not gossip' which was 'reassuring in a small community'

b) Being able to visit clients in their homes which they felt instilled hope

c) Minimising stigma associated with having a mental health problem. The lay workers heard from their clients that as they were already connected with public health work (rather than psychiatry) and carried out home visits routinely as part of their work on public health promotion and that it was not stigmatising for clients with *kufungisisa *(thinking too much) to be visited.

d) The structured 'talk therapy' helped them to monitor the progress and challenges that clients were facing.

e) Breaking down the problems into specific and manageable steps

f) Giving feedback to clients.

In the focus group, the lay workers reported several case histories of their clients. These included the following:

i) A female client who had been to the bench with a score of 12/14 on the SSQ at baseline and subsequently received 2 home visits described the lay health workers as 'bringing peace' in her home, and 'less agitation' from her partner. Her score dropped to 7/14 after six sessions.

ii) A female client with an SSQ of 11/14 dropped to 6/14 after five sessions which included a home visit after she presented with being unable to come to terms with her HIV status.

iii) A female senior member of the local protestant church described the home visits as 'hope for those of us who are unable to open up in a church congregation about our HIV status'. Her score went down to 5/14 from 10/14 after 6 sessions.

## Discussion

This is the first example of lay health workers in Africa delivering a low intensity mental health intervention, using locally adapted tools, for common mental disorders in primary care. We have shown that it is feasible for lay workers to deliver this intervention for depression and common mental disorders, and that recruitment to the intervention from primary care, community agencies and self-referral was also feasible (Figure 2). The treatment appeared acceptable to the community and the lay workers were able to integrate the intervention into their routine work. Preliminary findings also show that the intervention is efficacious in reducing psychological morbidity, with a drop in score of nearly 5 points on the 14-item psychological outcome scale after 3-6 sessions, and efficacy proportional to the number of sessions attended. Over half of those who participated had presented with a problem related to HIV.

Chance does not seem a likely explanation for our finding as the significance value for the drop in score after 3-6 sessions was at p < 0.01 level. Bias may explain some of the results in that women and married participants were more likely to participate than to decline or to be lost to follow-up and those with lower education were more likely to be lost to follow-up than to participate. However, overall, the response rate of 88% was extremely high so it appears unlikely that bias is playing a major role in explaining the results. Measurement error is also unlikely to explain the findings. The Shona Symptom Questionnaire was developed using optimal cross-cultural methods and has been validated against an international diagnostic interview with most of those scoring at or above the recommended cut-off having mixed depression and anxiety or pure depression using ICD criteria [4].

We do not have a comparison group from the same study who did not receive the intervention. However, a prospective study in primary care in Harare showed that a mean drop in score of 4.7 (sd 6.3) on the SSQ was associated with recovery from 'case' to 'non-case' and with significantly less disability [20] (see Table 3 of the Patel paper). These authors further report that those who experienced a drop in score of 4 or more points on the SSQ were more likely to self-report an improvement in health than those who remained at case-level on the SSQ. Our crude mean drop in score of 4.8 points thus appears to represent a meaningful drop in score indicating efficacy of the Friendship Bench intervention. Furthermore, our finding that drop in score was significantly correlated with the number of sessions attended, even after adjusting for baseline SSQ score, adds weight to our assertion that the intervention appears to be efficacious. In our pilot, 34% remained cases at 6-8 weeks follow-up after the intervention, whereas in the Patel et al study [20], where there was no specific intervention, 48% of primary health care attenders remained cases.

The quantitative findings are supported by the lay workers evaluation. All of them rated at least half of their clients as benefiting from problem-solving therapy with 7/10 rating 'more than half' of their clients benefiting from the intervention. Themes that emerged from qualitative work support the argument that implementing this intervention through an existing public health intervention and by mature women with a position of trust in the community, helps explain its apparent efficacy. The lay workers-or 'grandmother health providers' are viewed as wise, confidential, authoritative and not prone to gossip. As the lay workers were already respected for their public health work, participants said they did not find it not stigmatising to be visited.

The intervention is theoretically closely linked to problem-solving therapy, which has been shown to be effective for depression and common mental health problems [12,13], together with an activity scheduling component [21]. It incorporates local adaptations that are integral to the routine work of the therapists who are culturally sanctioned lay health workers, known and respected as 'grandmother health providers'. For instance, the inclusion of Christian prayer for 15 minutes during 1 or 2 of the 6 sessions was part of the existing practice of the lay workers and it would have been inappropriate to remove that normal practice. While there is no evidence from randomised controlled trials that prayer is an effective treatment for depression in Christians, there is some suggestion from non-randomised studies with small samples that religious activities may benefit depression [22].

In 1994 we showed that major barriers to up-scaling mental health care in this setting include lack of supervision, and lack of recording systems for common mental disorders. We provided evidence that problems in improving primary mental health care may be less with the attitudes (or even the training) of primary care staff and more with bureaucratic limitations such as the inadequacy of the diagnostic codes, absence of mental health supervision, lack of protocol for following-up CMD patients, lack of medicines, and lack of incentives to see patients with CMD [23]. The Friendship Bench has managed to address some of these challenges, especially through making use of lay workers and providing a system for them of peer and nurse-led supervision, with an available step up to specialist care which has been used in less than 10% of cases screening positive for CMD. An emphasis on local concepts and terms helped to reduce stigma of mental disorders. The local knowledge of the lay workers facilitated linkage with two local income-generating projects (peanut butter making; recycling) for those in most financial need.

Our decision to use problem-solving therapy was anchored in earlier evidence that *kufungisisa *and common mental disorders were associated with everyday social and health problems [16,24], and that the community trusted the lay health workers to aid them in resolving these problems using culturally accepted methods, which sometimes included prayer. This is consistent with the traditionally accepted rationale for using problem-solving therapy [19]. We found that those who used a greater number of sessions benefited more. Thus in future work it will be important to optimise adherence to the intervention and to follow-up clients. Given the value found in Western settings of written materials for clients in low-intensity psychological treatments, we wish to develop these for clients in Zimbabwe, with the aim of increasing the efficacy of the therapy. We will also add training in more collaborative structured approaches to activity scheduling for clients who remain depressed despite problem-solving therapy.

Of the 320 participants in this pilot, just over half had presented with an HIV-related problem. There is need to evaluate whether treatment for depression might improve physical health outcomes such as medication adherence in those who have depression co-morbid with physical illness [25].

With the large treatment gap that currently exists in low and middle income countries for mental health care, lay workers may be able to play a pivotal role [26]. In Zimbabwe, earlier work has shown the feasibility of using lay workers to prevent mother to child transmission of HIV, and to screen for psychological morbidity [5,27,28]. While there is evidence supporting the effectiveness of task-shifting in HIV [29], immunization, malaria prevention, and management of upper respiratory infections [30], ours is an important study given the dearth of evidence on lay workers addressing depression and common mental health problems in Africa.

Limitations of the study include the short follow-up period of 6 weeks. Also, the observed drop in SSQ score after six sessions of problem-solving therapy was not controlled for potential confounding factors such as socio-economic position [31]. The fundamental limitation to this study is the absence of a comparison group receiving 'usual care' or a placebo intervention, if one could be found. Furthermore, the lay workers were not observed during the course of their work and what they did in practice could have differed from their training. The low level of attrition among the participants is unusual; however, this could be attributed to the short follow up period, the close proximity of participants to the local study area, and the ability of the lay workers and research nurse to physically follow up participants in the community, and is consistent with high follow-up rates found in previous research in Harare [20]. The City of Harare Health Department continued the Friendship Bench after the pilot. In the 14 months from January 2008 to February 2009, 2348 clients had visited the Friendship Bench with 973 having received the problem-solving intervention. In view of this it is imperative to rigorously test this intervention.

## Conclusion

We have found preliminary evidence that lay primary health care workers can deliver locally adapted problem-solving therapy in Harare, Zimbabwe and that this can be associated with a meaningful reduction in symptoms of depression and common mental disorders. The problem solving therapy was integrated into the routine work load of the community based lay workers whose roles include supporting people living with HIV and carrying out health promotion activities. There is need to carry out appropriately powered randomised controlled trials to test if this task-shifting mental health intervention is effective compared to usual care in reducing psychological symptoms and also in improving physical health outcomes in those who have depression co-morbid with physical illness.

## Competing interests

The authors declare that they have no competing interests.

## Authors' contributions

DC was responsible for study design, data collection, writing manuscript and analysis of the data. PM and LK responsible for study design and review of second draft. FC reviewed second draft. RA assisted with analysing data and editing manuscripts.

MA developed Multiple Symptoms Card, made comments on first draft of manuscript, responsible for second draft of manuscript, contributed to revisions following referees comments and to writing of final draft. All authors read and approved the manuscript.

## Funding

No external funding

## Pre-publication history

The pre-publication history for this paper can be accessed here:

http://www.biomedcentral.com/1471-2458/11/828/prepub
